# Nuclear Shield: A Multi-Enzyme Task-Force for Nucleus Protection

**DOI:** 10.1371/journal.pone.0014125

**Published:** 2010-12-10

**Authors:** Raffaele Fabrini, Alessio Bocedi, Valentina Pallottini, Lorena Canuti, Michele De Canio, Andrea Urbani, Valeria Marzano, Tommaso Cornetta, Pasquale Stano, Anna Giovanetti, Lorenzo Stella, Antonella Canini, Giorgio Federici, Giorgio Ricci

**Affiliations:** 1 Department of Chemical Sciences and Technologies, University of Rome Tor Vergata, Rome, Italy; 2 Department of Internal Medicine, University of Rome Tor Vergata, Rome, Italy; 3 Department of Biology, University of Roma Tre, Rome, Italy; 4 Department of Biology, University of Rome Tor Vergata, Rome, Italy; 5 S. Lucia Research Institute IRCCS, Rome, Italy; 6 Institute of Radiation Protection, ENEA-CR Casaccia, Rome, Italy; St George's University of London, United Kingdom

## Abstract

**Background:**

In eukaryotic cells the nuclear envelope isolates and protects DNA from molecules that could damage its structure or interfere with its processing. Moreover, selected protection enzymes and vitamins act as efficient guardians against toxic compounds both in the nucleoplasm and in the cytosol. The observation that a cytosolic detoxifying and antioxidant enzyme *i.e.* glutathione transferase is accumulated in the perinuclear region of the rat hepatocytes suggests that other unrecognized modalities of nuclear protection may exist. Here we show evidence for the existence of a safeguard enzyme machinery formed by an hyper-crowding of cationic enzymes and proteins encompassing the nuclear membrane and promoted by electrostatic interactions.

**Methodology/Principal Findings:**

Electron spectroscopic imaging, zeta potential measurements, isoelectrofocusing, comet assay and mass spectrometry have been used to characterize this surprising structure that is present in the cells of all rat tissues examined (liver, kidney, heart, lung and brain), and that behaves as a “nuclear shield”. In hepatocytes, this hyper-crowding structure is about 300 nm thick, it is mainly formed by cationic enzymes and the local concentration of key protection enzymes, such as glutathione transferase, catalase and glutathione peroxidase is up to seven times higher than in the cytosol. The catalytic activity of these enzymes, when packed in the shield, is not modified and their relative concentrations vary remarkably in different tissues. Removal of this protective shield renders chromosomes more sensitive to damage by oxidative stress. Specific nuclear proteins anchored to the outer nuclear envelope are likely involved in the shield formation and stabilization.

**Conclusions/Significance:**

The characterization of this previously unrecognized nuclear shield in different tissues opens a new interesting scenario for physiological and protection processes in eukaryotic cells. Selection and accumulation of protection enzymes near sensitive targets represents a new safeguard modality which deeply differs from the adaptive response which is based on expression of specific enzymes.

## Introduction

In eukaryotic cells different types of biologic machineries contribute to protect DNA from molecules that could damage its structure or interfere with its processing. The nuclear envelope is a first important mechanical barrier that opposes the interaction of toxic compounds with the genetic material [1]. A second one is represented by specific protection enzymes and molecules (*i.e.* glutathione, vitamin A, C and E) able to eliminate many dangerous compounds. A third protection mechanism is formed by specific transcription factors mediated pathways [2]. Among the many toxic and dangerous compounds for the nucleus, a prominent killer role is due to compounds that produce oxidative (ROS), nitrosative (RNS) and alkylative stress. Catalase (CAT), glutathione peroxidase (GPX) (scavengers of H_2_O_2_) and superoxide dismutase (SOD) (which eliminates HO_2_^•^ radicals) are the most important antioxidant enzymes that counteract in many cells the killer activity of ROS. Recently an active antioxidant role has been described for heme oxygenase-2 in specific cell lines [3], [4] and for DNA polimerase iota, an enzyme which has intranuclear localization [5]. Glutathione transferases (GSTs), a superfamily of enzymes grouped in at least eight gene-independent classes in mammals, are also involved in the cell protection against alkylating compounds and organic peroxides. These enzymes catalyze the conjugation of glutathione (GSH) to the electrophilic centre of toxic alkylating compounds [6] and the Alpha class isoenzymes display a selenium-independent glutathione peroxidase activity [6]. We have also demonstrated that GSTs is involved in the cell defence against excess nitric oxide (NO) sequestering this free radical in a harmless iron complex bound to the active site [7]. Thus GST represents a multifunctional enzyme involved in the protection against ROS, RNS as well as against electrophilic agents. While this enzyme can be up-regulated in case of electrophilic or ROS stress [8], [9], the intracellular concentrations of CAT and GPX in various tissues cannot be increased in case of oxidative stress conditions [10], [11]. Despite the absence of a general adaptive response, a permanent optimization of the defence power could be reached by increasing the local concentration of protection enzymes near sensible intracellular targets like the nucleus. Possible existence of this novel defence strategy is suggested by a few observations: a curious presence of GSTs near the nucleus has been reported many years ago in immunohistochemical and non-aqueous cell fractionation studies [12], [13], [14]. More recently, we have observed a relevant accumulation of Alpha class GSTs near the nuclear membrane of the rat hepatocytes [15], a phenomenon revealed by the expedient of avoiding exogenous salts or buffers during the purification of the nuclear fraction [15]. The presence of salts or buffers, usually employed for nuclear preparations, easily detached these proteins from the membrane, a finding suggesting a predominant contribution of electrostatic interactions in this binding [15]. Importantly, the use of a specific fluorescent probe for GST signalled its perinuclear accumulation even in intact cells [15]. However, all these previous studies did not verify if other key protection enzymes beside GSTs are present. The possibility that an unrecognized more complex enzyme organization near the nucleus may exist is a stimulating proposal worthy to be investigated.

The present study explores the following possibilities: a) that beside GSTs, additional protection enzymes may be associated to the nuclear envelope, b) that this phenomenon may not be restricted to hepatocytes, c) that it has a specific protection function. We demonstrate here for the first time that a surprising selected multi-enzyme machinery is present near the nuclear envelope of cells from many tissues forming a sort of enzyme task-force which contributes to DNA protection.

## Results

### First evidence and physical characterization of the nuclear shield

**Figure 1:**
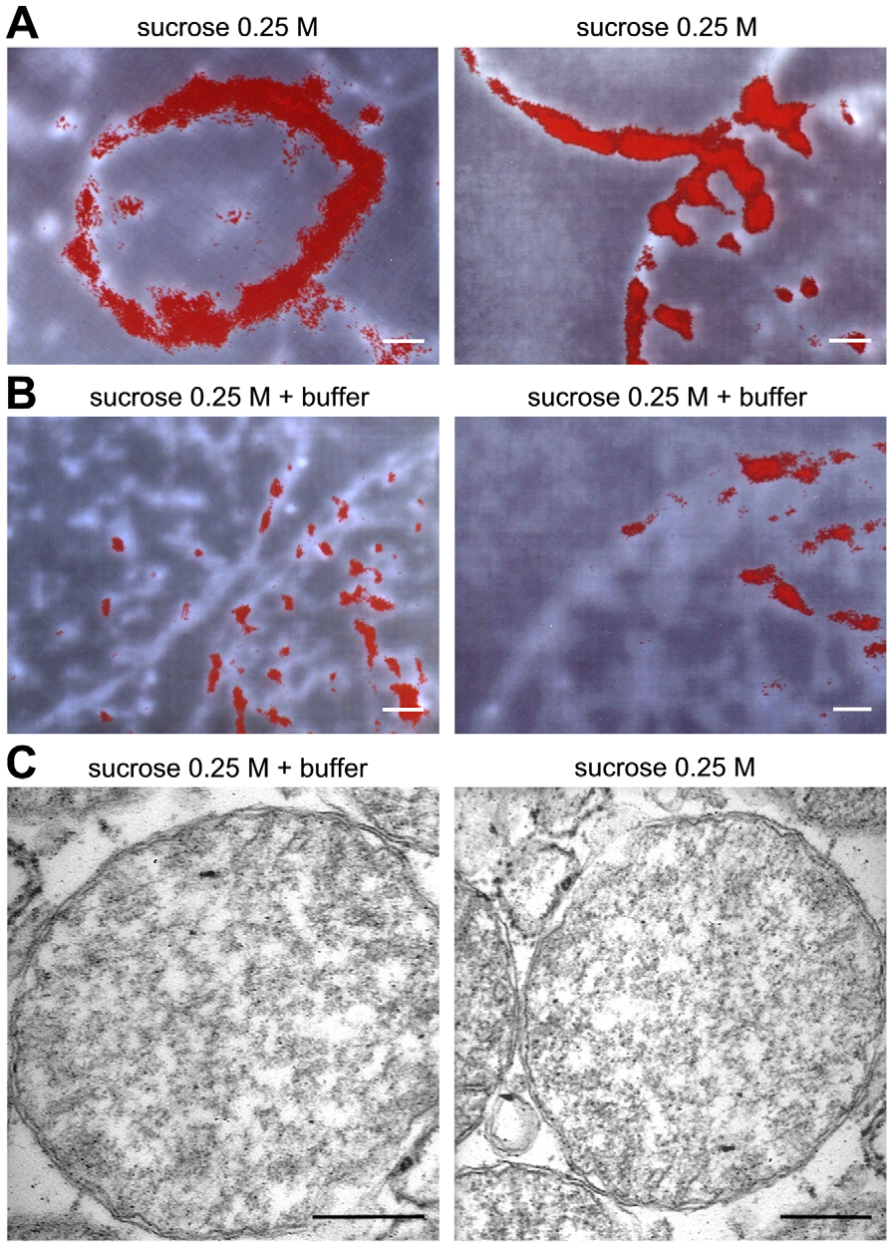
ESI experiments on shielded and de-shielded rat liver nuclei. (A) ESI of shielded nuclei. Images were obtained as described in Materials and Methods. The average of thickness of the red area showing the protein shield is 300±70 nm. Scale bar, 0.3 µm. (B) ESI of de-shielded nuclei. Scale bar, 0.3 µm. (C) TEM micrographs of shielded and de-shielded nuclei. Samples were treated and stained as reported in Materials and Methods. Scale bar, 0.3 µm.

In a first experimental approach, nuclei were purified in the absence of exogenous salts, and the proteins bound electrostatically to the nuclear membrane were detached by increasing the ionic strength (see Materials and Methods). Specifically, in rat liver 2.7 mg of nuclear bound proteins per gram of fresh tissue were extracted (Table 1), including, as expected, the Alpha class GSTs (see below). This amount represents about 3% of all cytosolic proteins. Nitrogen electron spectroscopic imaging (ESI) of nuclei purified in the absence of exogenous salts confirmed the presence of a thick protein structure surrounding the nuclear membrane, which appears like a “nuclear shield” and almost vanishes upon mild salt treatment (Figure 1, A and B). Statistical examination of ESI images at varying cut sections is consistent with an estimated thickness of the shield of about 300±70 nm. Given that the nuclear membrane available is 0.022 m^2^/g of liver [15], [16] (see Materials and Methods), the shield region has a protein density of about 0.4 g/cm^3^, a value twice as high as the one in the cytosolic milieu (∼0.2 g/cm^3^), but only half as much as the one in a protein crystal (∼1 g/cm^3^) [17]. In other words, this structure, rather than a compact multilayer [15], may be represented as a region of perinuclear hyper-crowding which is undetectable by means of standard morphological microscopy (Figure 1C).

**Table 1:** Nuclear shield proteins.

Organ	nuclear shield proteins (mg/g of tissue)	cytosolic proteins (mg/g of tissue)	membrane area/g of tissue (m^2^/g)	nuclear shield proteins/membrane area (mg/m^2^)
LIVER	2.7±0.4	85±5	0.022*	120±20
KIDNEY	2.6±0.3	70±6	0.027±0.001	100±10
HEART	3.8±0.2	31±5	0.032±0.001	119±7
LUNG	5.0±0.2	60±4	0.019±0.001	260±20
BRAIN	2.7±0.3	24±7	0.051±0.002	54±6

*Data from ref. [15].

### Zeta Potential measurements and nuclear shield re-formation

**Figure 2:**
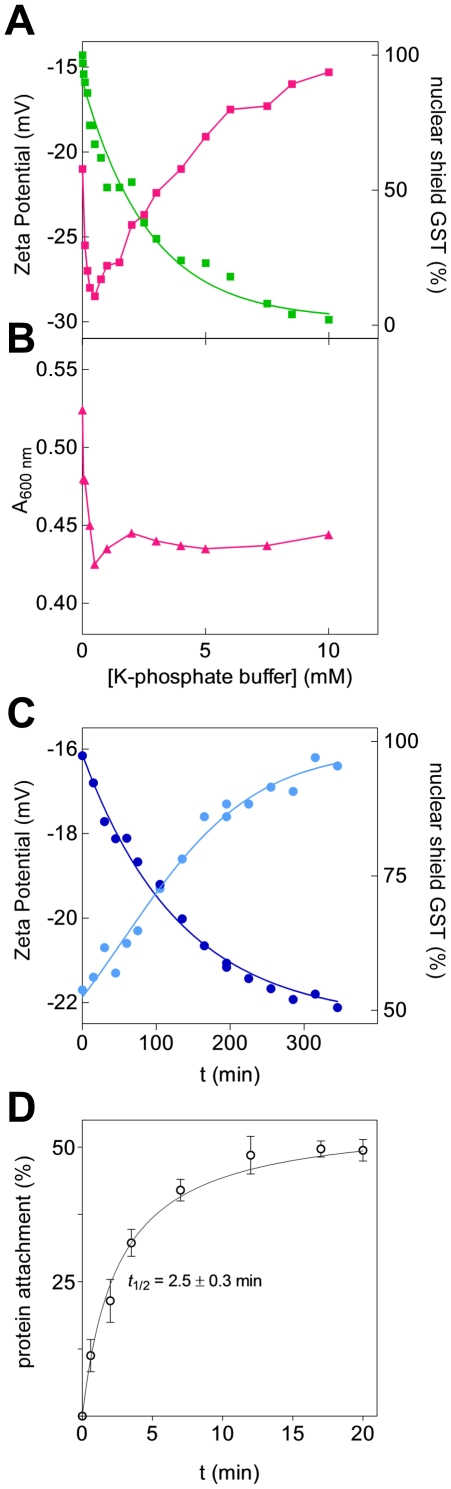
Zeta potential and shield re-formation. Shielded nuclei were diluted with 20 volumes of 0.25 M sucrose. (A) Zeta potential changes (*pink squares*) and GST detachment (*green squares*) due to the addition of potassium phosphate buffer, pH 7.4. Zeta potential perturbation at very low buffer concentration (below 1 mM) is due to a nuclear disaggregation process as suggested in (B) by a similar perturbation of light scattering at 600 nm (*pink triangles*). (C) Time dependent zeta potential changes and nuclear shield GST detachment in 0.25 M sucrose. Shielded nuclei suspended in 0.25 M sucrose were incubated at 25°C. At variable times the amount of GST released in solution (*blue circles*) and zeta potential (*light blue circles*) were measured. (D) Kinetic of shield re-formation. De-shielded nuclei in 0.25 M sucrose were divided into aliquots; each aliquot was resuspended in rat liver cytosolic fraction and incubated at 25°C. At fixed times, the mixture was centrifuged. The re-formation of the shield was measured either on the basis of protein content that can be detached with 50 mM NaCl. All experiments were performed in triplicate. Error bars, s.d.

Zeta potential, a sensitive function of the interface nature of suspended particles [18], [19], added further details on the protein organization of the shield. At increasing ionic strength, the change in zeta potential of isolated nuclear fractions parallels the GST detachment and proceeds without apparent discontinuity (Figure 2A), supporting the view that the different proteins forming the shield are mixed homogeneously. The zeta potential perturbation observed at very low ionic strength is likely due to a nuclear disaggregation process as suggested by the light scattering analysis (Figure 2B). We observed that a spontaneous and partial change of the zeta potential, accompanied by a parallel detachment of about 50% of the shield proteins, occurs even without addition of salts by simply incubating a dilute nuclear fraction in 0.25 M sucrose (Figure 2C). On the other hand, de-shielded nuclei extensively washed with 0.25 M sucrose are still able to re-constitute about 50% of the original nuclear shield when incubated with a cytosolic fraction. The process is fast but not instantaneous showing a *t*_1/2_ of 2.5 minutes (Figure 2D). The observed time dependent and spontaneous detachment of the nuclear shield (up to 50%) in 0.25 M sucrose suggests that the shield, as it appears immediately after nuclei preparation, could not be an artefact due to the use of sucrose. Obviously, the possibility that positively charged proteins present in the cytosol may be linked to a negative counterpart (*i.e.* proteins or phospholipid layers) depends on the relative competition between the cationic proteins and other positively charged ions (mainly K^+^). Thus, the relative concentration of these cationic objects drives protein attachment or detachment. The modality employed to prepare the nuclear fraction in 0.25 M sucrose does not perturb the reciprocal concentration of all cationic competitors and thus it appears a correct procedure to visualize specific electrostatic interactions like those which promote the nuclear shield. In addition, the existence of an artefact should be signalled by an increase in the shield when the relative amount of sucrose (compared to the cytosolic volume) is increased. Conversely, protein content of the nuclear shield remained almost unchanged when the nuclei were prepared from liver homogenate under different dilution conditions (*i.e.* 1∶3, 1∶6, 1∶10 and 1∶20 gram of tissue/ml of 0.25 M sucrose) (data not shown). Other previous evidences confirm the real existence of the shield; the perinuclear accumulation of GST has been demonstrated in intact cells (thus without sucrose) by using a specific fluorescent probe for this enzyme [15] and a similar evidence has been obtained using immunostaining procedures [12], [13], [14].

### Proteins of the nuclear shield display peculiar acid-base properties

**Figure 3:**
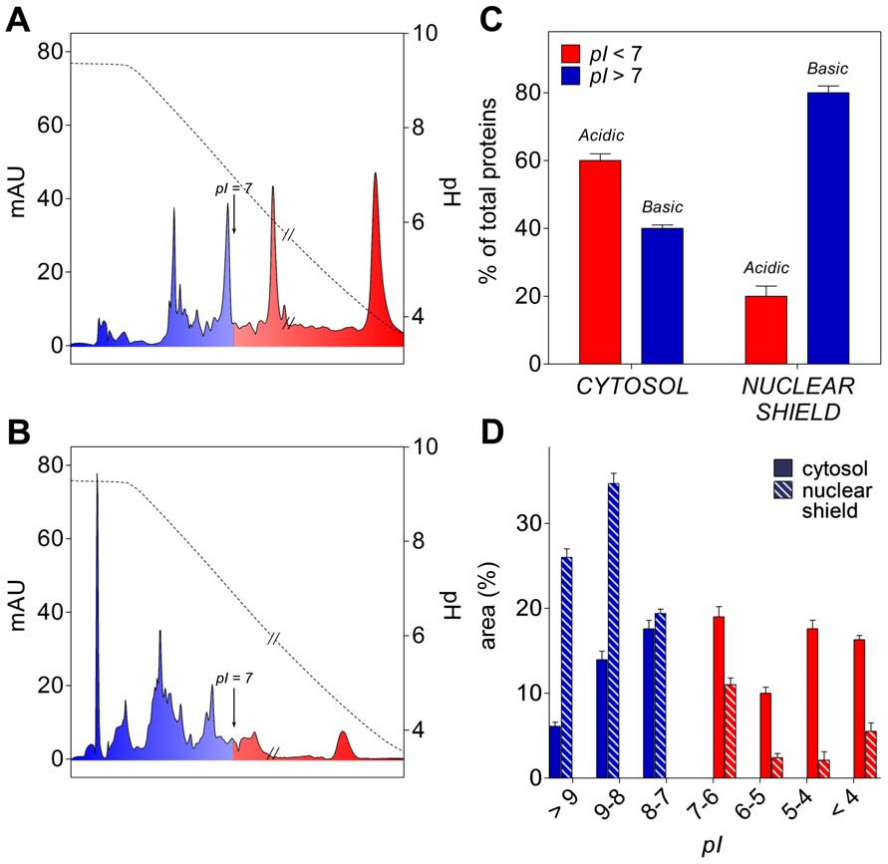
Isoelectrofocusing experiments. (A) Combined chromatogram of the cytosolic proteins obtained from two runs at different pH ranges (*i.e.* pH 9.0–6.0 and pH 7.0–4.0). The double slash shows the connection zone of the two chromatograms. Blue area indicates the basic proteins and red area the acidic proteins. Dotted line represents the pH gradient. (B) Combined chromatogram of the nuclear shield proteins. (C) Acid-base properties of nuclear shield and cytosolic proteins. (D) Percent of acidic and basic proteins grouped in discrete *pI* ranges as derived by the experiments shown in A and B.

We next examined whether the protein composition of the nuclear shield is similar to the one of the cytosolic fraction or rather only selected proteins are enriched in this perinuclear region. Isoelectric focusing experiments first suggested that this structure is formed prevalently by positively charged proteins (80%) while, in agreement with previous observations [20], the cytosol is populated predominantly by acidic proteins (60%) (Figure 3, A–C). Furthermore, a more stringent analysis of the basic region of the chromatograms indicated that the cationic proteins of the shield, grouped in discrete *pI* ranges, display a different distribution when compared to the cytosolic pool with an evident selection of the proteins with higher *pI* values (*pI*>8.0) (Figure 3D). The average *pI* of all shield proteins is ∼8.0 while the average *pI* of the cytosol is ∼6.0, a value close to that reported in previous studies (*pI*∼5.5) [21].

### Key antioxidant enzymes are present in the nuclear shield

**Figure 4:**
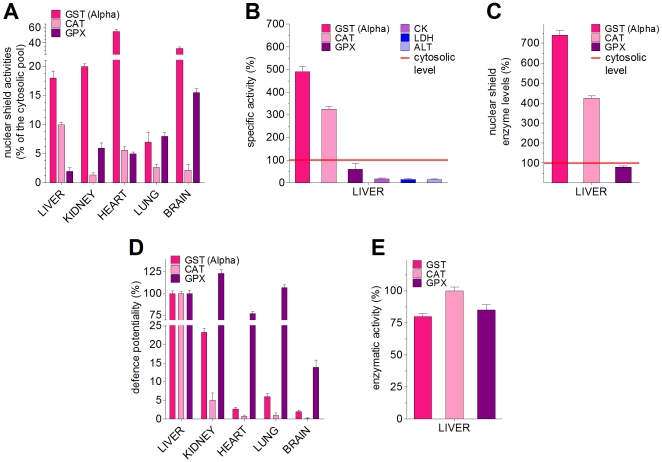
Quantification of antioxidant enzymes in the nuclear shield. Activity values reported in (A–E) are the means of 5 independent tissue preparations. Calculations in (A and C) were made assuming as reference the activity of the cytosolic Alpha GST found in distinct rat tissues (see Materials and Methods). (A) Nuclear shield activities of GST, CAT, GPX reported as percentages of the corresponding cytosolic activities. Error bars, s.d. (B) Specific activities of the antioxidant enzymes (GST, CAT and GPX) and non-antioxidant enzymes (CK, LDH and ALT) in the shield of rat liver. Specific activities of the corresponding cytosolic enzymes are taken as 100%; error bars, s.d. (C) Nuclear shield GST, CAT, and GPX concentrations compared with their cytosolic levels taken as 100%. A cytosol volume of 2630 µm^3^ for each hepatocyte and a shield volume of 67 µm^3^ (0.3 µm×222 µm^2^) were assumed for calculations [53]. Errors, s.d. (D) “Defense potentiality” of the nuclear shield. GST, CAT, and GPX activities found in the nuclear shield and normalized for nuclear membrane area (U/m^2^ of membrane area) of liver, kidney, heart, lung, and brain (from Table 2) were compared to those of liver taken as 100%. Error bars, s.d. (E) Catalytic activity of GST, CAT and GPX measured when they are free in solution (100%) or packed in the nuclear shield (see Materials and Methods); error bars, s.d.

The identification of specific enzyme activities in the nuclear shield could be diagnostic to clarify the physiological role of this structure. Beside the Alpha class GSTs – a well known efficient barrier against alkylating compounds, organic peroxides and nitric oxide [15], [22] – we detected significant amounts of additional anti-oxidant enzymes such as CAT and GPX (Table 2 and Figure 4A). In the nuclear shield all these enzymes have specific activities (U/mg of total proteins) similar and even higher than those found in the cytosol (five times higher for GST and three times for CAT) (Figure 4B) and increased local concentrations (seven times higher for GST and four times for CAT) (Figure 4C). The normalization of the activities of these enzymes to the corresponding nuclear membrane area is an additional parameter here termed “defence potentiality” of the shield (Table 2 and Figure 4D). For comparison, we examined the presence, in the nuclear shield, of enzymes that do not have a specific “protective role”, such as L-lactate dehydrogenase (LDH), L-alanine amino transferase (ALT), and creatine kinase (CK); these proteins are scarcely present in the shield and display very much lower specific activities than in the cytosol (Figure 4B). Curiously, the CAT identified in the shield is a peculiar cationic form (*pI* = 7.7) that is not found in the cytosol where a few anionic forms (*pI* = 5.8÷6.2), also present in large amounts in peroxisomes, are the predominant isoenzymes [23]. CAT is encoded by a single gene and its theoretical *pI* value, calculated on the basis of the amino acid composition, is 7.5 (see Materials and Methods) close to the one of the isoenzyme found in the shield, while the acidic cytoplasmic form is the result of post-translational modifications [24]. The shield contains only traces of an additional anti-oxidant enzyme *i.e.* SOD but its presence in this region could be pleonastic due to its cytosolic and nucleoplasmic localization [25]. Notably, the catalytic activities of GST, CAT and GPX are very similar when these enzymes are packed into the nuclear shield or free in solution (Figure 4E), demonstrating that active site accessibility and functional flexibility are not impaired by the increased protein density. Mass spectrometry analysis of the nuclear shield extract and of the cytosol disclosed additional details. Beside a clear confirmation of the prominent selection of cationic proteins in the shield (Figure S1, A and B), this approach indicated that, in addition to GST, CAT and GPX, many other enzymes contribute to the nuclear shield. Keeping in mind that mass spectrometry analysis allows to identify only proteins that display proper fragmentation patterns and volatility properties (*e.g.* GPX, whose activity was unambiguously identified, could not be detected either in the shield or in the cytosol), 78 distinct proteins have been identified in the shield, 39 of which are almost exclusive of this structure (Table S1 and S2). Of note, 72% of the shield proteins have, in their native oligomeric structures, molecular masses higher than the cut-off of the nuclear pores (kDa = 40÷45) [26], [27], indicating that they are unlikely to represent a contamination due to nuclear proteins (Table S2 and Figure S1C). Beside the postulated antioxidant role inferred from the observed enrichment of GST, CAT, and GPX, the nuclear shield may also have additional functions as hinted from the variegated enzyme mesh identified by mass spectrometry approach. In this respect it is worth pointing out that typical “metabolic enzymes” such as malate dehydrogenase and glyceraldehyde-3-phosphate dehydrogenase, identified in the nuclear shield, have been shown to be also part of transcriptional complexes and implicated in transcription regulation [28], [29].

**Table 2:** Antioxidant enzymes of the nuclear shield.

organ	cytosolic activity (U/g of tissue)				nuclear shield activity (U/g of tissue)			shield defense potentiality* (U/m^2^ of membrane area)		
	GST	GST (Alpha)**	CAT	GPX	GST (Alpha)†	CAT	GPX	GST(Alpha)†	CAT	GPX
LIVER	90±10	39±4	29000±5000	14±2	7±2	3000±600	0.28±0.04	300±80	140000±30000	13±2
KIDNEY	12±4	9±3	15000±2000	8±2	1.8±0.6	200±50	0.4±0.1	70±20	7000±2000	15±4
HEART	2.9±0.8	0.4±0.1	600±100	6±1	0.25±0.09	34±8	0.3±0.1	8±3	1100±200	9±3
LUNG	10±3	5±1	800±100	4±1	0.35±0.06	26±9	0.26±0.07	18±3	1400±500	14±4
BRAIN	6±2	0.9±0.3	370±50	0.6±0.1	0.3±0.1	10±3	0.09±0.03	6±2	200±60	1.8±0.6

Activities of GST, CAT and GPX were measured as reported in Materials and Methods. Values reported are the means of 5 independent tissue preparations. The standard deviation of the five different replicas are reported for each measurement. The units of the cytosolic GSTs (first column) are the sum of the activity contributions of all enzyme isoforms.

*“Defense potentiality” is defined as the amount of enzyme unit normalized per membrane area.

**Units of cytosolic Alpha class GST were calculated from previous studies (see Materials and Methods).

† GST units in the shield of various tissues were tentatively ascribed to Alpha class GST as occurs for the liver [15].

### DNA protection

**Figure 5:**
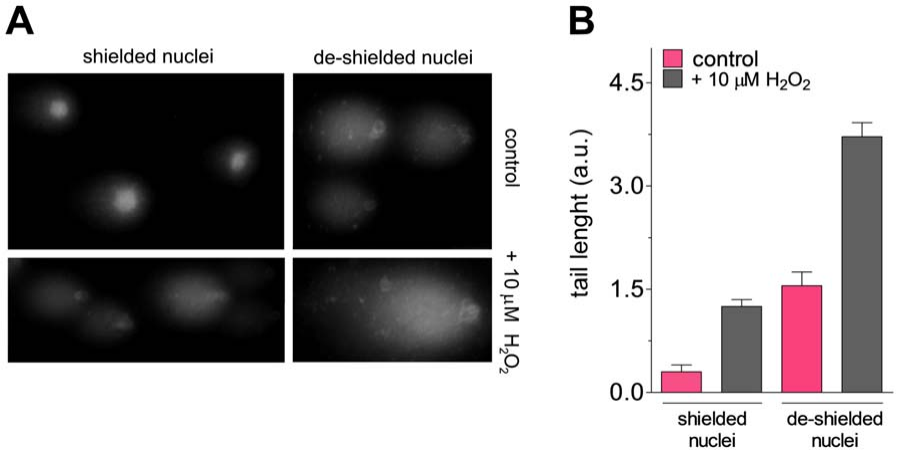
Protection of DNA from oxidative damage in the presence or absence of the nuclear shield. (A) Comet assay on shielded and de-shielded nuclei exposed to 10 µM H_2_O_2_ for three minutes. Experimental details are reported under Materials and Methods. (B) Statistical analysis of comet assay. Error bars, s.d.

Whatever possible additional roles played by such a variegated enzymatic composition of the shield, the clear enrichment of DNA-protective enzymes (Figure 4C) suggests for this newly identified structure a role as a guardian of genomic integrity. Comet assay, allowing the assessment of DNA breaks, performed on shielded and de-shielded nuclei, showed a direct evidence that the nuclear shield represents an efficient protection barrier for the genetic material, both in the presence and in the absence of oxidizing compounds (Figure 5A). A quantification of the damage produced by H_2_O_2_ on DNA has been obtained by measuring the tail length (Figure 5B). Furthermore, a clear indication that perinuclear Alpha GSTs may act as an efficient trap even against non-oxidizing toxic compounds like nitric oxide derivatives has been described previously in intact hepatocytes treated with NO donors [7].

### Nuclear shield compositions vary in cells of different tissues

This protein structure is not restricted to the liver cells but it is also present in the perinuclear regions of cells of tissues as diverse as kidney, heart, lung and brain (Table 1, Table 2 and Figure 4A). The most and the less populated nuclear shields were found in the lung and in the brain, respectively (Table 1). This difference might be related to the fact that lung cells are more exposed to exogenous toxic compounds and, conversely, the brain is a protected tissue by virtue of the hematoencephalic barrier. Interestingly, each tissue shows different and characteristic levels of antioxidant enzymes in the shield; for example, while the liver shield has a relevant amount of CAT and little GPX, the opposite it is true in the kidney, lung and brain (Figure 4A).

### Nuclear proteins are the electrostatic counterpart for the cationic shield

**Figure 6:**
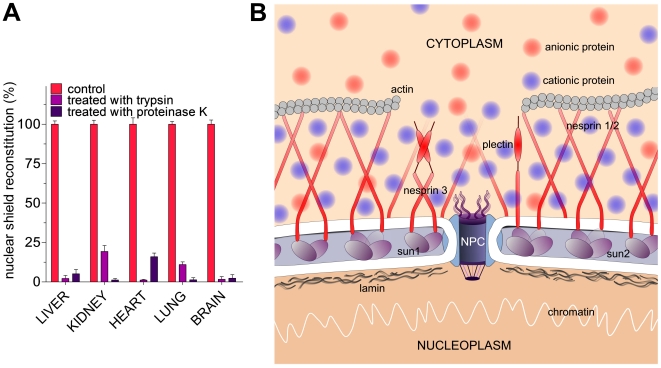
Proteolysis experiments and tentative pictorial representation of the nuclear shield. (A) Effect of proteolysis by trypsin and proteinase K on the nuclear shield reconstitution (see Materials and Methods). Error bars, s.d. (B) Tentative pictorial representation of nuclear shield on the outer nuclear membrane, also based on the models shown in refs. [32], [33]. Proportions between anionic and cationic proteins in the shield and in the cytosol shown in the picture are those obtained experimentally.

Only the nuclear membrane displays propensity to form the protein shield. Membranes of different intracellular organelles, such as mitochondria, microsomes and lysosomes, lack this enzymatic protection [15]. Given that all intracellular membranes have similar, albeit non identical, lipid composition, it is unlikely that subtle differences may be responsible for the unique presence of the shield on the nuclear membrane. We speculate that some negatively charged proteins, exclusively present in the nuclear membrane and firmly bound to the envelope, may provide the necessary electrostatic potential to attract and stabilize the protein shield. Proteolytic experiments support this hypothesis; incubation of de-shielded nuclei with trypsin or protease K completely inhibits the re-formation of the nuclear shield (Figure 6A).

## Discussion

For the first time a particular enzyme-network is identified which is electrostatically associated to the outer nuclear membrane. In the last years many studies have been performed to detail composition and role of the inner and outer nuclear envelopes and their enzymatic equipments. For example, nuclear pores and other integral membrane protein complexes (belonging to the inner membrane) have been found to play a fundamental role in the dynamic organization of the genome, positioning in DNA repair, recombination and stability [30]. On the other hand, recent findings point to important structural roles for nesprins and plectins, giant rod-like proteins anchored exclusively to the outer nuclear membrane [31]. These proteins stretch out into the cytosol and are involved in the nucleus positioning. The nuclear shield described in the present study implements the functions correlated to the outer nuclear membrane. The existence in different rat cells of a perinuclear multi-enzyme structure mainly formed by selected cationic proteins including key protection enzymes (Figure 3 and Table 2) indicates that this particular region may act as guard against oxidative damage. This peculiar finality can be only revealed on the basis of classical biochemical studies instead of immunocytochemical assays that cannot verify the enzymatic competence of the detected enzymes. Indeed, antibody works made in the past [12], [13], [14] clearly indicated the accumulation of GST in the perinuclear region but they were unable to assess any biological activity. The presence of active forms of GST, CAT and GPX, as determined in this paper, designs this perinuclear region a sort of hyper-filter where the detoxifying power is enhanced up to seven times with respect to the cytosol (Figure 4C). A simple comet assay visualizes an increased DNA protection against ROS favoured by this structure (Figure 5). Additional evidences point to a specific finality of the shield and not to a casual enzyme assemblage. For example, this structure appears more prominent in tissues more exposed to toxic compounds (lung) while it is less populated in the highly protected brain (Table 1). Furthermore, in tissues where CAT is abundant, GPX (which has similar detoxifying activity against H_2_O_2_) must be redundant and, accordingly, it is scarcely represented in the shield (Table 2). Obviously, the protection role may be just one of many other possible functions inherent in this structure. Beside CAT, GST and GPX, the large variety of different enzymes that composes the shield (Table S2) represents an unexplored mine for future investigations in this direction. We also note that most of the shield enzymes have molecular masses higher than the one allowing the entrance into the nucleus (Figure S1C) and thus this machinery may be a clever system to approach these enzymes to the chromosomes.

The biochemical and physical characterization of this novel cellular structure outlines a new level of cell organization, mediated by weak, relatively unspecific, electrostatic interactions, that somewhat contrasts a picture of the cell delineated by well organized organelles interspersed in a relatively homogeneous milieu. The particular protein density of the shield (only two times higher than in the cytosol) as calculated through ESI measurements, allows to assimilate this structure to an hyper-crowding which cannot be detected by classical microscopy (Figure 1C). This may explain why its presence remained masked until now. Interestingly, the lack of shield re-formation after proteolytic treatment of the nucleus (Figure 2D) indicated that some negatively charged proteins (specifically present on the outer nuclear envelope) could play a crucial role in the shield formation. Only a few membrane proteins may be indicated as possible shield scaffold. Among them, nesprin-1, nesprin-2 and plectin are giant proteins (ranging from 2800 to 6900 residues) showing rod-like structures about 300–400 nm long [31] (a length similar to the shield thickness), and exclusively present as a sort of negatively charged network on the outer nuclear membrane surface [32], [33]. These proteins are involved in the nucleus positioning and display conspicuous net negative charge *i.e.* about −70 for plectin, −100 for nesprin-1 and −240 for nesprin-2 (see Materials and Methods) that may represent a good electrostatic counterpart for the positively charged proteins of the nuclear shield. A tentative pictorial model of this structure is shown in Figure 6B.

If the nuclear shield is a functionally important structure, as proposed here, genetic alterations that reduce its thickness or modify its enzyme composition should affect the protective role and possibly result in pathological phenotypes. Intriguingly, many neurodegenerative pathologies like Alzheimer, Parkinson, Huntington and amyotrophic lateral sclerosis are characterized by typical aggregations of misfolded or damaged proteins in the perinuclear region termed aggresomes [34]. The possibility that such structures alter the protective nuclear shield described in this study is just one of the hypotheses that must be verified in the immediate future.

## Materials and Methods

### Reagents

All reagents used in this study were from Sigma-Aldrich Inc. (St. Louis, USA) and used without further purification. Trypsin and Protease K (Sigma-Aldrich) employed in the proteolytic experiments were from bovine pancreas (11,400 U/mg) and from *Tritirachium album* (13.1 U/mg), respectively.

### Animals

Male *Wistar* rats were anaesthetised with sodium pentobarbital (50 mg/kg body weight, injected *i.p.*) before rapid killing by cervical dislocation minimizing sufferings and subsequent liver, kidney, heart, lung and brain dissections. Experiments were carried out with the approval by the Ethic Committee of the University of Roma Tre in accordance to the ethical guidelines for animal research of the Italian Ministry of Health (permit number: 246/H10) D.Lvo 116/92.

### Nuclei preparations

After perfusion with 0.25 M sucrose and heparine to remove blood, different tissues (liver, kidney, heart, lung and brain) from male rats were excised, minced and homogenized in a teflonglass Potter Elvehjem homogenizer, in 0.25 M sucrose (10 ml per gram of tissue). After a brief centrifugation at 300×*g* (3 min) to remove unbroken cells and periplasmic membranes, the homogenate was centrifuged at 1000×*g* for 10 min to isolate the nuclear fraction [15]. The resulting supernatant, centrifuged at 100,000×*g* for 30 min, represents the “cytosolic extract”. The nuclear pellet was washed three times with 10 ml of 0.25 M sucrose and re-suspended in 10 ml of 0.25 M sucrose. This suspension represents the “shielded” nuclei fraction. It contains less than 2% of contaminating structures as judged by microscopy analysis. Even less contamination was observed by measuring marker enzymes of cytosolic, mitochondrial, lysosomal and microsomal origin [15]. De-shielded nuclei were obtained by incubating the shielded nuclei (coming from 1 gram of fresh tissue) with 50 mM NaCl (or 10 mM potassium phosphate buffer, pH 7.4). After centrifugation at 10,000×*g* for 5 min, 4°C, the pellet was re-suspended in 10 ml of 0.25 M sucrose. The total protein content of cytosolic fraction and nuclear shield fraction were determined by the method of Lowry [35].

### Nuclear membrane surface

Nuclear membrane area of cell from different rat tissues were calculated on the basis of the total phospholipidic content. Phospholipids were determined according to literature protocols [36], [37], [38]. The extraction step started from 800 µl of a nuclear suspension of different tissues prepared as above described. In a glass tube 4 ml of a solution CHCl_3_/MeOH 2∶1 v/v were mixed with the nuclear sample, vortexed for one min and centrifuged at 2,000 rpm for 2 minutes. The aqueous phase was discarded. Chromatographic controls were made by using a TLC slice of silica-gel POLYGRAM SIL UV 254 (0.25 mm gel with fluorescence probe), eluted with a solution CHCl_3_/MeOH/H_2_O/NH_3_ - 25% 65/30/4/2 v/v. The organic phase was mixed with 1 ml of NaCl 0.9% at 0°C, vortexed and centrifuged at 2,000 rpm for 2 minutes. The washing aqueous phase was discarded and the organic phase was stored at −20°C. To quantify the phospholipidic content, 80 µl of organic phase were evaporated (110°C, 15 min), then 70 µl of H_2_SO_4_/HClO_4_ 1∶1 v/v were added and the sample was heated at 240°C for 30 minutes. 1.6 ml of ascorbic acid/ammonium molibdate 0.83%/0.2% were added, vortexed and incubated at 45°C for 30 minutes. The blue color due to the complex formed was quantified spectrophotometrically at 820 nm. The calibration curve derived from a standard solution of 1 mM KH_2_PO_4_.

For quantitative analysis, the total membrane surface area of a single rat liver hepatocyte is assumed 110,000 µm^2^ [16]. Considering that 1 gram of fresh liver contains about 10^8^ cells and that the outer nuclear membrane is 0.2% of the total hepatocyte membranes, it results that the available outer nuclear surface area is about 0.022 m^2^ per gram of fresh liver. The membrane area/g of tissue for kidney, lung, heart, and brain was determined by evaluating the phospholipidic content as reported above and by comparing this value with the phospholipidic content found in rat liver.

### Nitrogen Electron Spectroscopic Imaging (ESI) and morphological microscopy

Shielded and de-shielded nuclei from rat hepatocytes suspended in 0.25 M sucrose were treated with a fixative solution containing 3% glutaraldehyde for six hours. Nuclei were post-fixed for 2 hours in 1% osmium tetroxide in the same buffer, dehydrated with ethanol, and embedded in epon 812 resin [39] (TAAB, England). Thin and ultra-thin (<40 nm) unstained sections were collected in uncoated 200 mesh copper grids and observed with a Zeiss CEM transmission electron microscope at 80 kV. Staining of the ultra-thin sections with lead citrate and uranyl acetate (usually performed to visualize better sub-cellular structures in transmission electron microscopy) was omitted to avoid the interference with the ESI and electron energy loss spectroscopy analyses. For the localization of N in the specimens, the electron spectroscopic images were taken at ΔE = 410 eV, just above the ionization edge (IE) of N (Nk ΔE = 401 eV), to detect the total N signal, and at ΔE = 377 eV, the pre-ionization edge (PIE) of N, as a reference carrying information on the background [39], [40]. The unit test area was circular with a variable diameter ranging from 1.3 to 8 mm, which was anyway smaller than the selected microscopic field and inversely proportional to the degree of microscopic magnification. Net N images were obtained by using a digital image analyzer with an interactive built analysis system: the N map obtained by subtracting the PIE from the IE images was recorded with a highly sensitive camera.

### Enzymatic assays and catalytic activities

GST, CAT, GPX, and SOD activities were assayed both in cytosolic and nuclear shield fractions following usual assay conditions [15], [41], [42], [43]. Cytosolic and nuclear shield fractions were treated with dithiothreitol (DTT) to preserve GPX enzymatic activity [42]. Enzymatic assays for CK, LDH, and ALT were carried out on a Modular P800 device (Roche) [44]. The enzymatic units of cytosolic GSTs are the sum of the activities of all enzyme isoforms. Conversely, as demonstrated previously for the liver nuclei [15], the activities of GSTs found in the shield of the different rat tissues can be related mainly to the Alpha class GSTs. The cytosolic Alpha GST abundances are calculated on the basis of previous studies on liver [45], kidney [46], heart [47], lung [48], and brain [49] GSTs.

Comparison of the catalytic activities of GST, CAT and GPX bound to the nuclear shield or free in solution was made on shielded nuclei aliquots (suspended in 0.25 M sucrose), in the absence or in the presence of 50 mM NaCl. Activities were measured by usual spectrophotometric methods [15], [41], [42] but in the absence of any buffer.

### Zeta potential measurements

Zeta potential values were obtained by using a Laser-Doppler microelectrophoresis using a Zetasizer 5000 instrument (Malvern, UK). Measurement cell was aligned before analysis by using reference latex beads (−50±5 mV) (Malvern, UK). Zeta potentials were calculated by the integrated Malvern proprietary software (v. 1.36) by using the Smoluchowsky model (F(ka) = 1.5). Reported values refer to the average of five independent measurements (variation coefficient ca. 5–10%). Shielded nuclei samples from rat liver in 0.25 M sucrose, kept constantly at 4°C, were treated with variable amounts of 0.1 M potassium-phosphate buffer, pH 7.4 (or of 1 M NaCl). The volume of the added buffer did not exceed 2.5% v/v. Samples were then poured into the measurement cell and measured after temperature equilibration at 25°C (about 1 minute). Preliminary experiments have shown that nuclei do not sediment significantly in such time interval.

### Nuclear shield re-formation

Experiment is illustrated in Scheme S1 (Supporting information). Nuclei from rat liver were de-shielded with 50 mM NaCl and washed three times with 0.25 M sucrose. The nuclear suspension was divided into aliquots, each aliquot was resuspended in rat liver cytosolic fraction and incubated at 25°C. At fixed times, the mixture was centrifuged. The re-formation of the shield was measured either on the basis of protein detached from the nuclei with 50 mM NaCl or, alternatively, by measuring the decrease of the total protein concentration of the cytosol after incubation with the de-shielded nuclei. Experiments were performed in triplicate.

### FPLC Chromatofocusing

Isoelectrofocusing experiments were performed on an AKTA Purifier system (Amersham Biosciences, Inc.), equipped with a pump system (P-900), spectroscopy unit (UV-900), pH-meter/conductimeter (pH/C-900), and a fraction collector (Frac-900). The chromatography runs were performed on a Mono P 5/200 GL (HR 5/20) column (Amersham Biosciences Inc.), with 1 ml/min flow rate. The pH range explored in two different chromatographic runs were from 9.0 to 6.0 using a start buffer ethanolamine-CH_3_COOH 0.025 M pH 9.4 and a Polybuffer 96-CH_3_COOH pH 6.0 (Amersham Biosciences, Inc.), and from 7.0 to 4.0 by a start buffer bis/Tris-Iminodiacetic acid 0.025 M pH 7.4 and a Polybuffer 74-Iminodiacetic acid pH 4.0 (Amersham Biosciences, Inc.). Rat hepatocyte cytosol extract and nuclear shield extract were loaded at a concentration of 0.3 mg/ml. The final chromatograms were recorded and analyzed by the software Unicorn 5.2 (Amersham Biosciences, Inc.).

### Theoretical *pI* calculations

Theoretical *pI* estimation and electrostatic calculations were performed at pH 7.0 by the Protein Calculator Server v. 3.3 at www.scripps.edu. Catalase *pI* estimation (accession number: AAB42378). Proteins selected for net charge calculations: plectin (accession number: CAA42169), nesprin-1 (accession number: AAL47053) and nesprin-2 (accession number: XP_001080795).

### Mass spectrometry analysis

Proteins derived from rat liver cytosolic fraction and nuclear shield fraction were precipitated with 50% ethanol, 25% methanol, 25% acetone and dissolved in 6 M urea and 100 mM Tris pH 7.9. After reduction with 10 mM DTT and alkylation with 20 mM iodacetamide, protein samples were digested 50∶1 (w/w) with sequence grade trypsin (Promega, Madison, WI, USA) at 37°C overnight. The reaction was stopped by adding a final concentration of 0.1% trifluoroacetic acid. Samples were diluted with 0.1% formic acid, 3% acetonitrile in water at a concentration of 0.36 µg/µl, and 0.72 µg of protein digestion were loaded on column for peptide separation. Peptides were trapped on a 5 µm Symmetry C18 trapping column 180 µm×20 mm (Waters Corp., Milford, MA, USA) and separated using a 175 min reversed phase gradient at 250 nl/min (3 to 40% acetonitrile in water over 125 min) on a nanoACQUITY UPLC™ System (Waters), utilizing a 1.7 µm BEH 130 C18 NanoEase™ 75 µm×25 cm nanoscale LC column (Waters). The lock mass ([Glu1]-Fibrinopeptide B/µl, 500 fmol/µl) was delivered from the auxiliary pump of the with a constant flow rate of 200 nl/min. The separated peptides were mass analyzed by a hybrid quadrupole orthogonal acceleration time-of-flight mass spectrometer (Q-Tof Premier™, Waters Corp.) directly coupled to the chromatographic system and programmed to step between low (4 eV) and high (15–40 eV) collision energies on the gas cell, using a scan time of 1.5 per function over 50–1990 m/z (Expression mode: data independent parallel parent and fragment ion analysis [50]). Continuum LC-MS data from three replicates experiments for both rat liver fractions were processed using the software ProteinLynx Global Server v2.3 (Waters). Protein identifications were obtained with the embedded ion accounting algorithm of the software [51] and searching on the UniProtKB/Swiss-Prot protein knowledgebase (release 57.15 of 02-March-10 containing 515203 sequence entries, with taxonomical restriction: *Rattus norvegicus*, 7483 sequence entries). The search parameters were: automatic tolerance for precursor ions and for product ions, minimum 3 fragment ions matched per peptide, minimum 7 fragment ions matched per protein, minimum 2 peptides matched per protein, 1 missed cleavage, carbamydomethylation of cysteine as fixed modification and oxidation of methionine as variable modification.

### Comet assay

Rat liver nuclei suspension in 0.25 M sucrose and the corresponding de-shielded nuclei were treated with H_2_O_2_ (10 µM) for three minutes. The assay was performed starting with 20 µl of nuclei suspension (∼10.000–20.000 nuclei) mixed with 180 µl of 1.7% low melting agarose in sucrose 0.25 M and immediately pipetted onto a frosted glass microscope slide pre-coated with a layer of 1% normal melting point agarose, prepared in PBS lacking Ca^2+^ and Mg^2+^. Three slides were prepared for each experimental point. The agarose was allowed to set at 4°C for the necessary time. After, slides were placed on a horizontal electrophoresis unit containing fresh buffer (1 mM EDTA, 300 mM NaOH pH 13.0). Electrophoresis was then conducted in fresh electrophoresis buffer (pH 13.0) for 15 min at 25 V and 300 mA (0.8 V/cm) at 4°C. Subsequently, the slides were gently washed in neutralization solution (0.4 M Tris-HCl, pH 7.5) for 5 min and fixed in 100% fresh methanol for 3 min. Slides were stained with 50 µl ethidium bromide (20 µg/ml) and covered with a coverslip. The main protocol for comet assay was based according by usual method [52]. Stained nucleoids were scored visually using a fluorescence microscope (Leica) equipped with a camera COHU (20× magnification). Three slides were analyzed for each experimental point and comet images on each slide were acquired using the ‘I.A.S.’ automatic image analysis software purchased from Delta Sistemi (Rome, Italy). The comet images were digitalized and a statistical assessment of tail length was conducted.

### Proteolysis experiments

The experiment is illustrated in Scheme S2 (Supporting information). Nuclei from one gram of five different rat tissues were de-shielded with 50 mM NaCl, washed three times with 10 ml of 0.25 M sucrose and resuspended in 0.25 M sucrose (10 ml). Two samples for each tissue (1 ml) were incubated for 2 hours at 25°C with trypsin and proteinase K (1.0 mg/ml final concentration). A third sample was incubated only with 50 mM NaCl, and a fourth sample taken as simple control (sample D). After incubation the samples were washed three times in 0.25 M sucrose. Proteolyzed and control nuclei from liver, kidney, heart, lung and brain were incubated with their corresponding cytosolic fractions for 20 minutes at 25°C, and then washed three times with 0.25 M sucrose. The amount of re-constituted shield was evaluated by measuring the proteins or Alpha GST detached by 50 mM NaCl.

## Supporting Information

Figure S1 Mass spectrometry analysis. (A) Theoretical *pI* distribution of the cytosolic and nuclear shield proteins identified by LC-MSE (see Materials and Methods). Statistical significance was calculated according to a nonparametric Wilcoxon-Mann-Whitney test. (B) Percent of total acidic and basic proteins in cytosol and nuclear shield. (C) Molecular masses of the native proteins found in the shield. *Green circles*: proteins with molecular masses higher than the cut-off value of nuclear pores. *Green circles with arrow*: proteins bound with protein complexes with higher molecular masses. *Pink circles*: proteins with molecular masses lower than cut-off value of nuclear pores.
(0.62 MB TIF)

Table S1 Proteins identified in cytosolic fraction.*
(0.13 MB DOC)

Table S2 Proteins identified in nuclear shield fraction.*^¶^
(0.11 MB DOC)

Scheme S1 (0.65 MB TIF)

Scheme S2 (0.41 MB TIF)
