# Supplementary figures and images for: Nuclear Shield: A Multi-Enzyme Task-Force for Nucleus Protection
Source: PLoS One. 2010 Dec 10;5(12):e14125. doi: 10.1371/journal.pone.0014125 (PMC3000810; doi:10.1371/journal.pone.0014125)

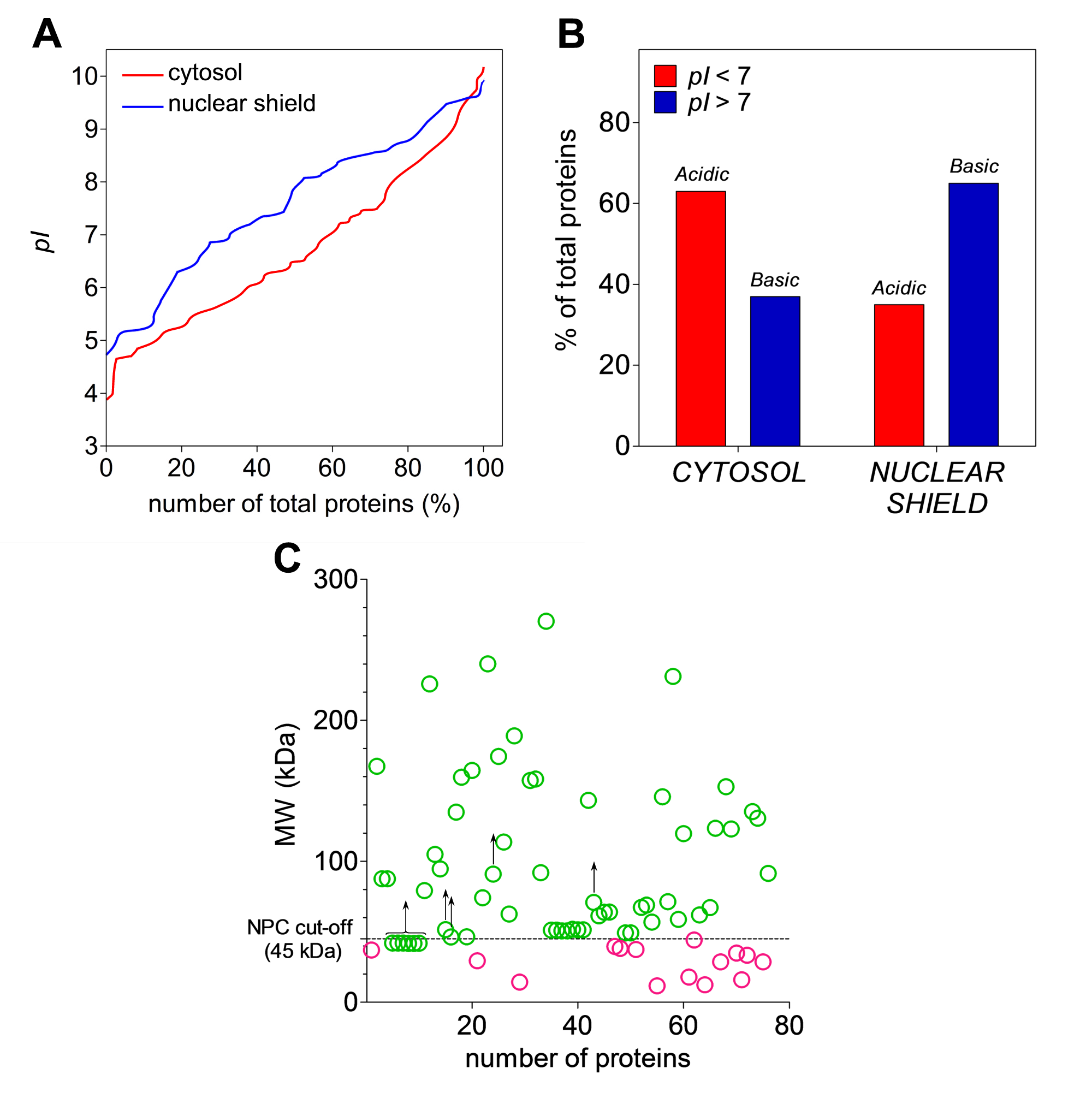

Supplement: Figure S1 — Mass spectrometry analysis. (A) Theoretical pI distribution of the cytosolic and nuclear shield proteins identified by LC-MSE (see Materials and Methods). Statistical significance was calculated according to a nonparametric Wilcoxon-Mann-Whitney test. (B) Percent of total acidic and basic proteins in cytosol and nuclear shield. (C) Molecular masses of the native proteins found in the shield. Green circles: proteins with molecular masses higher than the cut-off value of nuclear pores. Green circles with arrow: proteins bound with protein complexes with higher molecular masses. Pink circles: proteins with molecular masses lower than cut-off value of nuclear pores. (0.62 MB TIF) [file pone.0014125.s001.tif]

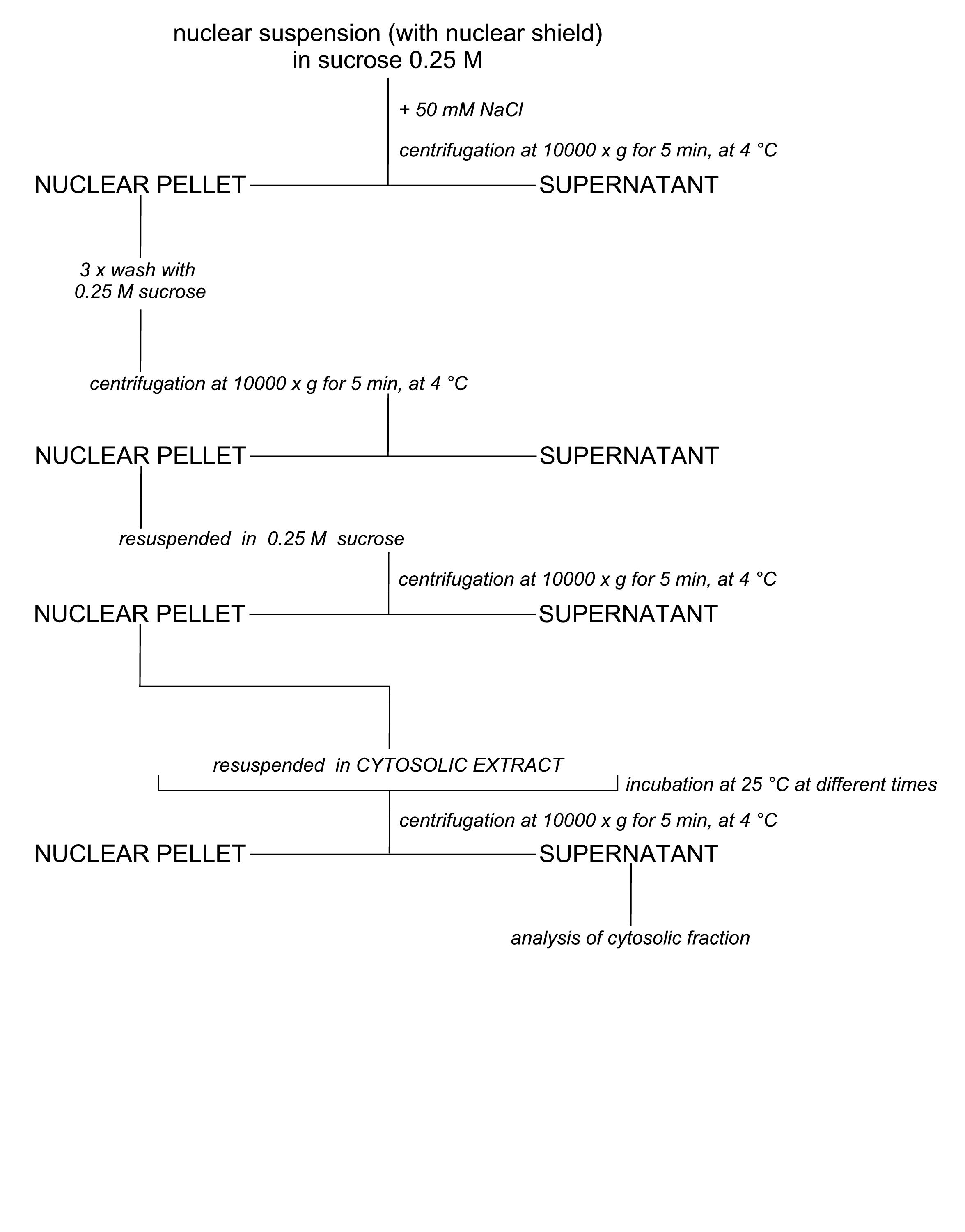

Supplement: Scheme S1 — (0.65 MB TIF) [file pone.0014125.s004.tif]

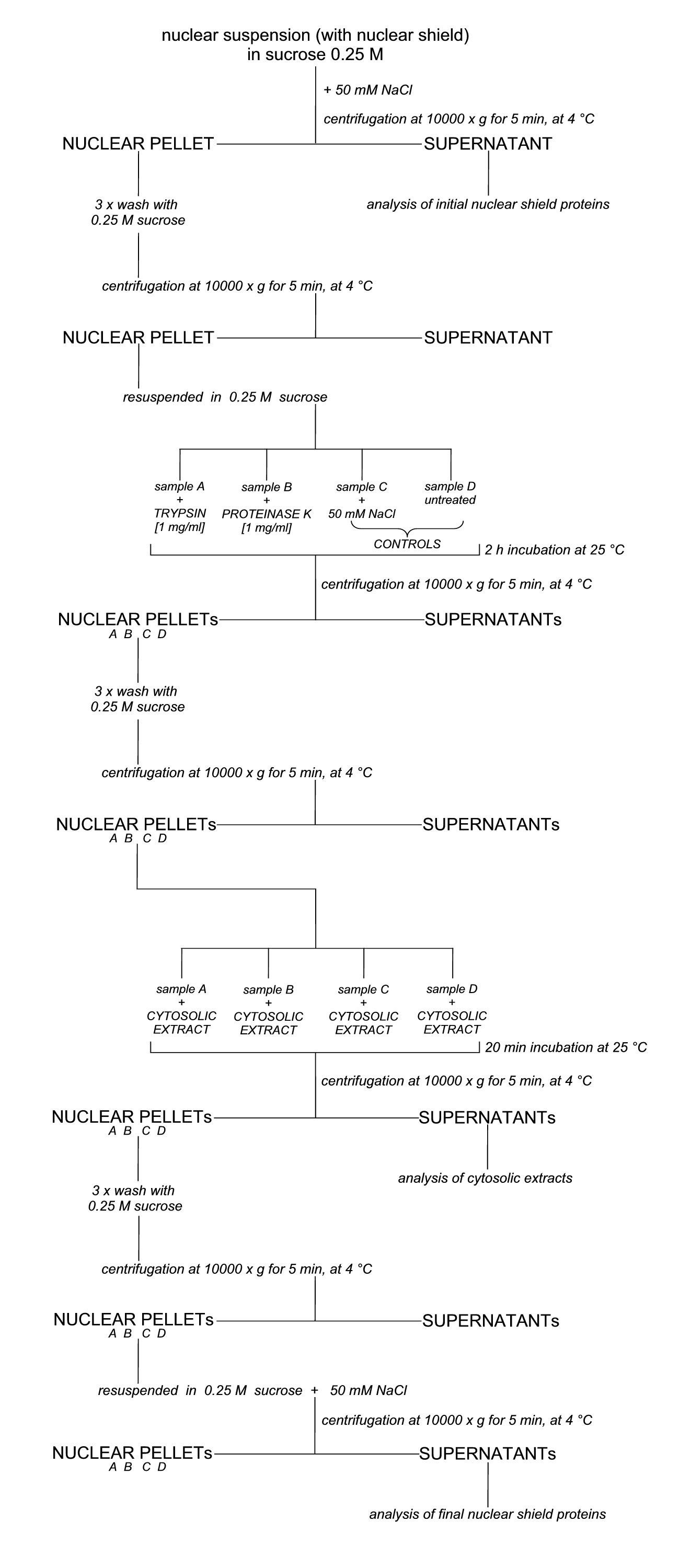

Supplement: Scheme S2 — (0.41 MB TIF) [file pone.0014125.s005.tif]
