# Supplementary material for: Nuclear Shield: A Multi-Enzyme Task-Force for Nucleus Protection
Source: PLoS One. 2010 Dec 10;5(12):e14125. doi: 10.1371/journal.pone.0014125 (PMC3000810; doi:10.1371/journal.pone.0014125)
Supplement: Table S1 — Proteins identified in cytosolic fraction.* (0.13 MB DOC) [file pone.0014125.s002.doc]

| **Table S1** Proteins identified in cytosolic fraction* | | | | | |  |
| --- | --- | --- | --- | --- | --- | --- |
|  | **Accession** ^†^  (UniProtKB/ Swiss-Prot) | **Description** | **MW** ^‡^ (Da) | ***pI*** ^‡^ |  | |
|  | P28037 | 10-formyltetrahydrofolate dehydrogenase | 99063 | 5.71 |  | |
|  | P23457 | 3-alpha hydroxysteroid dehydrogenase | 37003 | 6.72 |  | |
|  | P46953 | 3-hydroxyanthranilate-3,4-dioxygenase | 32561 | 5.47 |  | |
|  | P21775 | 3-ketoacyl-CoA thiolase A peroxisomal | 43805 | 8.20 |  | |
|  | P07871 | 3-ketoacyl-CoA thiolase B peroxisomal | 43792 | 8.20 |  | |
|  | P31210 | 3-oxo-5-beta steroid-4-dehydrogenase | 37354 | 6.17 |  | |
|  | P32755 | 4-hydroxyphenylpyruvate dioxygenase | 45083 | 6.30 |  | |
|  | Q9JLJ3 | 4-trimethylaminobutyraldehyde dehydrogenase | 53618 | 6.57 |  | |
|  | P68035 | Actin alpha cardiac muscle 1 | 41991 | 5.07 |  | |
|  | P68136 | Actin alpha skeletal muscle | 42023 | 5.07 |  | |
|  | P62738 | Actin aortic smooth muscle | 41981 | 5.08 |  | |
|  | P60711 | Actin cytoplasmic 1 | 41709 | 5.14 |  | |
|  | P63259 | Actin cytoplasmic 2 | 41765 | 5.16 |  | |
|  | P63269 | Actin gamma enteric smooth muscle | 41849 | 5.16 |  | |
|  | P11030 | Acyl-CoA binding protein | 10021 | 9.39 |  | |
|  | Q64640 | Adenosine kinase | 40108 | 5.64 |  | |
|  | P10760 | Adenosylhomocysteinase | 47507 | 6.06 |  | |
|  | P06757 | Alcohol dehydrogenase 1 | 39619 | 8.10 |  | |
|  | P51652 | Aldo keto reductase family 1 member C18 | 37275 | 5.86 |  | |
|  | Q6AYQ2 | Aldo keto reductase family 1 member C21 | 36111 | 7.21 |  | |
|  | P04764 | Alpha enolase | 47098 | 6.14 |  | |
|  | P09034 | Argininosuccinate synthase | 46466 | 7.74 |  | |
|  | P13221 | Aspartate aminotransferase cytoplasmic | 46399 | 6.82 |  | |
|  | P15429 | Beta enolase | 46984 | 7.23 |  | |
|  | O09171 | Betaine homocysteine S-methyltransferase 1 | 44947 | 7.85 |  | |
|  | Q68FT5 | Betaine homocysteine S-methyltransferase 2 | 39903 | 6.17 |  | |
|  | Q63276 | Bile acid-CoA amino acid N-acyltransferase | 46435 | 7.06 |  | |
|  | P62161 | Calmodulin | 16826 | 3.88 |  | |
|  | P07756 | Carbamoyl phosphate synthase ammonia mitochondrial | 164474 | 6.31 |  | |
|  | P14141 | Carbonic anhydrase 3 | 29412 | 6.99 |  | |
|  | P04762 | Catalase | 59719 | 7.16 |  | |
|  | P22734 | Catechol O-methyltransferase | 29578 | 5.27 |  | |
|  | P80254 | D dopachrome decarboxylase | 13124 | 6.11 |  | |
|  | P06214 | Delta aminolevulinic acid dehydratase | 36008 | 6.33 |  | |
|  | P62630 | Elongation factor 1 alpha 1 | 50082 | 9.34 |  | |
|  | P52844 | Estrogen sulfotransferase isoform 1 | 35485 | 5.70 |  | |
|  | P52845 | Estrogen sulfotransferase isoform 2 | 35341 | 5.45 |  | |
|  | P49889 | Estrogen sulfotransferase isoform 3 | 35392 | 5.44 |  | |
|  | P49890 | Estrogen sulfotransferase isoform 6 | 35279 | 5.55 |  | |
|  | P02692 | Fatty acid binding protein liver | 14263 | 8.57 |  | |
|  | O88618 | Formimidoyltransferase cyclodeaminase | 58876 | 5.71 |  | |
|  | P19112 | Fructose 1,6-bisphosphatase 1 | 39584 | 5.41 |  | |
|  | P00884 | Fructose bisphosphate aldolase B | 39593 | 8.43 |  | |
|  | P25093 | Fumarylacetoacetase | 45946 | 6.73 |  | |
|  | P07323 | Gamma enolase | 47110 | 4.84 |  | |
|  | P00502 | Glutathione S-transferase alpha 1 | 25590 | 9.16 |  | |
|  | P04903 | Glutathione S-transferase alpha 2 | 25542 | 9.17 |  | |
|  | P04904 | Glutathione S-transferase alpha 3 | 25303 | 9.10 |  | |
|  | P46418 | Glutathione S-transferase alpha 5 | 25331 | 8.81 |  | |
|  | P04905 | Glutathione S-transferase Mu 1 | 25897 | 8.35 |  | |
|  | P08010 | Glutathione S-transferase Mu 2 | 25685 | 7.22 |  | |
|  | P08009 | Glutathione S-transferase Yb 3 | 25664 | 7.17 |  | |
|  | P04797 | Glyceraldehyde-3-phosphate dehydrogenase | 35805 | 8.08 |  | |
|  | Q9ESV6 | Glyceraldehyde-3-phosphate dehydrogenase testis specific | 46677 | 7.84 |  | |
|  | P13255 | Glycine N-methyltransferase | 32528 | 7.22 |  | |
|  | P55063 | Heat shock 70 kDa protein 1 like | 70505 | 5.83 |  | |
|  | Q07439 | Heat shock 70 kDa protein 1A 1B | 70142 | 5.47 |  | |
|  | P63018 | Heat shock cognate 71 kDa protein | 70827 | 5.20 |  | |
|  | P82995 | Heat shock protein HSP 90 alpha | 84761 | 4.74 |  | |
|  | P34058 | Heat shock protein HSP 90 beta | 83229 | 4.78 |  | |
|  | P14659 | Heat shock related 70 kDa protein 2 | 69598 | 5.34 |  | |
|  | P01946 | Hemoglobin subunit alpha 1 2 | 15318 | 8.03 |  | |
|  | P02091 | Hemoglobin subunit beta 1 | 15969 | 8.25 |  | |
|  | P11517 | Hemoglobin subunit beta 2 | 15972 | 9.20 |  | |
|  | P62959 | Histidine triad nucleotide binding protein 1 | 13768 | 6.41 |  | |
|  | Q5XIC3 | Hsp90 co chaperone Cdc37 like 1 | 38290 | 4.97 |  | |
|  | O35952 | Hydroxyacylglutathione hydrolase mitochondrial | 34087 | 7.60 |  | |
|  | P20760 | Ig gamma 2A chain C region | 35163 | 7.53 |  | |
|  | P41562 | Isocitrate dehydrogenase NADP cytoplasmic | 46704 | 6.57 |  | |
|  | P04642 | L-lactate dehydrogenase A chain | 36427 | 8.37 |  | |
|  | O88989 | Malate dehydrogenase cytoplasmic | 36460 | 6.15 |  | |
|  | Q05982 | Nucleoside diphosphate kinase A | 17181 | 5.91 |  | |
|  | P19804 | Nucleoside diphosphate kinase B | 17271 | 7.32 |  | |
|  | Q497B0 | Omega amidase NIT2 | 30681 | 7.08 |  | |
|  | P04550 | Parathymosin | 11552 | 3.95 |  | |
|  | P10111 | Peptidyl prolyl cis-trans isomerase A | 17862 | 8.27 |  | |
|  | Q9R063 | Peroxiredoxin 5 mitochondrial | 22164 | 8.83 |  | |
|  | O35244 | Peroxiredoxin 6 | 24802 | 5.54 |  | |
|  | P04176 | Phenylalanine 4 hydroxylase | 51789 | 5.69 |  | |
|  | P31044 | Phosphatidylethanolamine binding protein 1 | 20788 | 5.38 |  | |
|  | P16617 | Phosphoglycerate kinase 1 | 44509 | 7.90 |  | |
|  | P25113 | Phosphoglycerate mutase 1 | 28813 | 6.79 |  | |
|  | P62963 | Profilin 1 | 14947 | 8.48 |  | |
|  | P97584 | Prostaglandin reductase 1 | 35695 | 8.08 |  | |
|  | O88767 | Protein DJ 1 | 19961 | 6.36 |  | |
|  | P61459 | Pterin 4 alpha carbinolamine dehydratase | 11992 | 6.33 |  | |
|  | P51156 | Ras related protein Rab 26 | 28185 | 9.75 |  | |
|  | Q03336 | Regucalcin | 33368 | 5.10 |  | |
|  | P52759 | Ribonuclease UK114 | 14294 | 8.59 |  | |
|  | P13444 | S-adenosylmethionine synthetase isoform type 1 | 43670 | 5.53 |  | |
|  | P18298 | S-adenosylmethionine synthetase isoform type 2 | 43688 | 5.90 |  | |
|  | Q8VIF7 | Selenium binding protein 1 | 52498 | 6.10 |  | |
|  | P12346 | Serotransferrin | 76345 | 6.99 |  | |
|  | P02770 | Serum albumin | 68686 | 6.05 |  | |
|  | P27867 | Sorbitol dehydrogenase | 38209 | 7.19 |  | |
|  | Q66X93 | Staphylococcal nuclease domain containing protein 1 | 101888 | 6.77 |  | |
|  | P17988 | Sulfotransferase 1A1 | 33884 | 6.40 |  | |
|  | P50237 | Sulfotransferase 1C1 | 35740 | 6.09 |  | |
|  | P07632 | Superoxide dismutase Cu,Zn | 15901 | 5.88 |  | |
|  | Q3B7D3 | Tectonic 2 | 77251 | 5.37 |  | |
|  | P11232 | Thioredoxin | 11665 | 4.60 |  | |
|  | P46462 | Transitional endoplasmic reticulum ATPase | 89292 | 4.95 |  | |
|  | P50137 | Transketolase | 67600 | 7.21 |  | |
|  | P48500 | Triosephosphate isomerase | 26831 | 6.99 |  | |
|  | P68370 | Tubulin alpha 1A chain | 50103 | 4.76 |  | |
|  | Q6P9V9 | Tubulin alpha 1B chain | 50119 | 4.76 |  | |
|  | Q6AYZ1 | Tubulin alpha 1C chain | 49905 | 4.79 |  | |
|  | Q5XIF6 | Tubulin alpha 4A chain | 49892 | 4.75 |  | |
|  | P85108 | Tubulin beta 2A chain | 49874 | 4.59 |  | |
|  | Q3KRE8 | Tubulin beta 2B chain | 49920 | 4.59 |  | |
|  | Q6P9T8 | Tubulin beta 2C chain | 49769 | 4.60 |  | |
|  | Q4QRB4 | Tubulin beta 3 chain | 50386 | 4.63 |  | |
|  | P69897 | Tubulin beta 5 chain | 49638 | 4.59 |  | |
|  | P50545 | Tyrosine protein kinase HCK | 59115 | 7.22 |  | |
|  | P62989 | Ubiquitin | 8559 | 7.67 |  | |

* Proteins present in cytosolic fraction of rat liver as described in Materials and Methods.

^†^ Accession numbers from UniProtKB/Swiss-Prot Release 57.15 of identified proteins.

^‡^ Theoretical MW and p*I* values.
