# Supplementary material for: Nuclear Shield: A Multi-Enzyme Task-Force for Nucleus Protection
Source: PLoS One. 2010 Dec 10;5(12):e14125. doi: 10.1371/journal.pone.0014125 (PMC3000810; doi:10.1371/journal.pone.0014125)
Supplement: Table S2 — Proteins identified in nuclear shield fraction.*¶ (0.11 MB DOC) [file pone.0014125.s003.doc]

**Table S2** Proteins identified in nuclear shield fraction*,¶

| **Accession** ^†^  (UniProtKB/ Swiss-Prot) | **Description** | **MW** ^‡^ (Da) | ***pI*** ^‡^ | **Native MW**  (Da) |
| --- | --- | --- | --- | --- |
| P23457 | 3-alpha hydroxysteroid dehydrogenase | 37003 | 6.72 | 37003 |
| **P13437** | **3-ketoacyl-CoA thiolase mitochondrial** | **41844** | **7.92** | **167376** |
| P21775 | 3-ketoacyl-CoA thiolase A peroxisomal | 43805 | 8.20 | 87610 |
| P07871 | 3-ketoacyl-CoA thiolase B peroxisomal | 43792 | 8.20 | 87584 |
| P68035 | Actin alpha cardiac muscle 1 | 41991 | 5.07 | > 41991 ^a^ |
| P68136 | Actin alpha skeletal muscle | 42023 | 5.07 | > 42023 ^a^ |
| P62738 | Actin aortic smooth muscle | 41981 | 5.08 | > 41981 ^a^ |
| P60711 | Actin cytoplasmic 1 | 41709 | 5.14 | > 41709 ^a^ |
| P63259 | Actin cytoplasmic 2 | 41765 | 5.16 | > 41765 ^a^ |
| P63269 | Actin gamma enteric smooth muscle | 41849 | 5.16 | > 41849 ^a^ |
| P06757 | Alcohol dehydrogenase 1 | 39619 | 8.10 | 79238 |
| **P11884** | **Aldehyde dehydrogenase mitochondrial** | **56452** | **6.67** | **225808** |
| **P07824** | **Arginase 1** | **34951** | **6.88** | **104853** |
| **P00507** | **Aspartate aminotransferase mitochondrial** | **47284** | **9.26** | **94568** |
| **Q91XJ1** | **Beclin 1** | **51524** | **4.70** | **> 51524 ^b^** |
| **P29067** | **Beta arrestin 2** | **46310** | **7.51** | **> 46310 ^c^** |
| O09171 | Betaine homocysteine S-methyltransferase 1 | 44947 | 7.85 | 134841 |
| Q68FT5 | Betaine homocysteine S-methyltransferase 2 | 39903 | 6.17 | 159612 |
| Q63276 | Bile acid-CoA amino acid N-acyltransferase | 46435 | 7.06 | 46435 |
| P07756 | Carbamoyl phosphate synthase ammonia mitochondrial | 164474 | 6.31 | 164474 |
| P14141 | Carbonic anhydrase 3 | 29412 | 6.99 | 29412 |
| **P18886** | **Carnitine O-palmitoyltransferase 2 mitochondrial** | **74063** | **6.93** | **74063** |
| P04762 | Catalase | 59719 | 7.16 | 238876 |
| **Q9JJ31** | **Cullin 5** | **90832** | **7.92** | **> 90832 ^d^** |
| **P18757** | **Cystathionine gamma lyase** | **43577** | **7.86** | **174308** |
| **Q63150** | **Dihydropyrimidinase** | **56778** | **6.81** | **113556** |
| **P13803** | **Electron transfer flavoprotein subunit alpha mitochondrial** | **34929** | **8.49** | **62616 ^e^** |
| **P14604** | **Enoyl-CoA hydratase mitochondrial** | **31496** | **8.16** | **188976** |
| P02692 | Fatty acid binding protein liver | 14263 | 8.57 | 14263 |
| **Q6GX84** | **Fidgetin like protein 1** | **74150** | **6.60** | **444900** |
| **P05065** | **Fructose bisphosphate aldolase A** | **39327** | **8.07** | **157308** |
| P00884 | Fructose bisphosphate aldolase B | 39593 | 8.43 | 158372 |
| P25093 | Fumarylacetoacetase | 45946 | 6.73 | 91892 |
| **P15431** | **Gamma aminobutyric acid receptor subunit beta 1** | **54037** | **8.94** | **270185** |
| P00502 | Glutathione S-transferase alpha 1 | 25590 | 9.16 | 51180 |
| P04903 | Glutathione S-transferase alpha 2 | 25542 | 9.17 | 51084 |
| P04904 | Glutathione S-transferase alpha 3 | 25303 | 9.10 | 50606 |
| P46418 | Glutathione S-transferase alpha 5 | 25331 | 8.81 | 50662 |
| P04905 | Glutathione S-transferase Mu 1 | 25897 | 8.35 | 51794 |
| P08010 | Glutathione S-transferase Mu 2 | 25685 | 7.22 | 51370 |
| P08009 | Glutathione S-transferase Yb 3 | 25664 | 7.17 | 51328 |
| P04797 | Glyceraldehyde 3-phosphate dehydrogenase | 35805 | 8.08 | 143220 |
| P63018 | Heat shock cognate 71 kDa protein | 70827 | 5.20 | > 70827 ^f^ |
| P01946 | Hemoglobin subunit alpha 1 2 | 15318 | 8.03 | 61272 |
| P02091 | Hemoglobin subunit beta 1 | 15969 | 8.25 | 63876 |
| P11517 | Hemoglobin subunit beta 2 | 15972 | 9.20 | 63888 |
| **Q6URK4** | **Heterogeneous nuclear ribonucleoprotein A3** | **39627** | **9.22** | **39627** |
| **Q9JJ54** | **Heterogeneous nuclear ribonucleoprotein D0** | **38168** | **7.90** | **38168** |
| **Q8VHV7** | **Heterogeneous nuclear ribonucleoprotein H** | **49157** | **5.64** | **49157** |
| **Q6AY09** | **Heterogeneous nuclear ribonucleoprotein H2** | **49262** | **5.85** | **49262** |
| **A7VJC2** | **Heterogeneous nuclear ribonucleoproteins A2 B1** | **37454** | **9.19** | **37454** |
| **P30823** | **High affinity cationic amino acid transporter 1** | **67222** | **5.58** | **67222** |
| **Q9WVK7** | **Hydroxyacyl coenzyme A dehydrogenase mitochondrial** | **34425** | **9.18** | **68850** |
| **P22791** | **Hydroxymethylglutaryl-CoA synthase mitochondrial** | **56875** | **8.79** | **56875** |
| P20760 | Ig gamma 2A chain C region | 35163 | 7.53 | - |
| **P01835** | **Ig kappa chain C region B allele** | **11593** | **4.78** | **11593** |
| P04642 | L-lactate dehydrogenase A chain | 36427 | 8.37 | 145708 |
| **P04636** | **Malate dehydrogenase mitochondrial** | **35660** | **8.82** | **71320** |
| **Q02253** | **Methylmalonate semialdehyde dehydrogenase acylating mitoc.** | **57770** | **8.25** | **231080** |
| **P11915** | **Non specific lipid transfer protein** | **58775** | **6.63** | **58775** |
| **P00481** | **Ornithine carbamoyltransferase mitochondrial** | **39860** | **9.44** | **119580** |
| P10111 | Peptidyl prolyl cis-trans isomerase A | 17862 | 8.27 | 17862 |
| **Q63716** | **Peroxiredoxin 1** | **22095** | **8.24** | **44190** |
| **Q9Z0V5** | **Peroxiredoxin 4** | **30988** | **6.19** | **61976** |
| **P83871** | **PHD finger like domain containing protein 5A** | **12396** | **8.36** | **12396** |
| **Q642A7** | **Protein FAM151A** | **67144** | **6.27** | **67144** |
| **Q9R189** | **Protein unc 13 homolog D** | **123376** | **6.19** | **123376** |
| P52759 | Ribonuclease UK114 | 14294 | 8.59 | 28588 |
| P27867 | Sorbitol dehydrogenase | 38209 | 7.19 | 152836 |
| **Q3T1I5** | **Sterol regulatory element binding protein 2** | **122898** | **8.40** | **122898** |
| **Q9WUW9** | **Sulfotransferase 1C2A** | **34836** | **7.17** | **34836** |
| P07632 | Superoxide dismutase Cu,Zn | 15901 | 5.88 | 15901 |
| **P24329** | **Thiosulfate sulfurtransferase** | **33385** | **7.85** | **33385** |
| P50137 | Transketolase | 67600 | 7.21 | 135200 |
| **Q62689** | **Tyrosine protein kinase JAK2** | **130501** | **7.01** | **130501** |
| P62989 | Ubiquitin | 8559 | 7.67 | - |
| **Q6P7Q0** | **UPF0516 protein C12orf72 homolog** | **28622** | **5.19** | **28622** |
| **Q75WE7** | **von Willebrand factor A domain containing protein 5A** | **91424** | **6.17** | **91424** |

* Proteins present in the nuclear shield of rat liver as described in Materials and Methods. In bold are reported characteristic proteins of the nuclear shield. The others are common proteins present in nuclear shield and cytosolic fractions.

^¶^ Few proteins reported maybe present only in traces: superoxide dismutase activity is very low (present paper) and glutathione S-transferase Mu cannot be detected neither by immunostaining nor by HPLC (see ref. [15]).

^†^ Accession numbers from UniProtKB/Swiss-Prot Release 57.15 of identified proteins.

^‡^ Theoretical MW and *pI* values.

^a^ Component of large polymeric protein complex.

^b^ Interacts with BCL2, AMBRA1 and many other proteins.

^c^ Forms oligomers and interacts with many other proteins.

^d^ Component of protein complexes.

^e^ Native MW of the heterodimer with beta subunit.

^f^ Component of protein complexes.
